# Physiological Importance of Hydrogen Sulfide: Emerging Potent Neuroprotector and Neuromodulator

**DOI:** 10.1155/2016/9049782

**Published:** 2016-06-20

**Authors:** Sandesh Panthi, Hyung-Joo Chung, Junyang Jung, Na Young Jeong

**Affiliations:** ^1^Department of Biomedical Science, Graduate School, Kyung Hee University, No. 26, Kyungheedae-ro, Dongdaemun-gu, Seoul 02447, Republic of Korea; ^2^Department of Anesthesiology and Pain Medicine, College of Medicine, Kosin University, No. 262, Gamcheon-ro, Seo-gu, Busan 49267, Republic of Korea; ^3^Department of Anatomy and Neurobiology, College of Medicine, Kyung Hee University, No. 26, Kyungheedae-ro, Dongdaemun-gu, Seoul 02447, Republic of Korea; ^4^Department of Anatomy and Cell Biology, College of Medicine, Dong-A University, No. 32, Daesingongwon-ro, Seo-gu, Busan 49201, Republic of Korea

## Abstract

Hydrogen sulfide (H_2_S) is an emerging neuromodulator that is considered to be a gasotransmitter similar to nitrogen oxide (NO) and carbon monoxide (CO). H_2_S exerts universal cytoprotective effects and acts as a defense mechanism in organisms ranging from bacteria to mammals. It is produced by the enzymes cystathionine *β*-synthase (CBS), cystathionine *ϒ*-lyase (CSE), 3-mercaptopyruvate sulfurtransferase (MST), and D-amino acid oxidase (DAO), which are also involved in tissue-specific biochemical pathways for H_2_S production in the human body. H_2_S exerts a wide range of pathological and physiological functions in the human body, from endocrine system and cellular longevity to hepatic protection and kidney function. Previous studies have shown that H_2_S plays important roles in peripheral nerve regeneration and degeneration and has significant value during Schwann cell dedifferentiation and proliferation but it is also associated with axonal degradation and the remyelination of Schwann cells. To date, physiological and toxic levels of H_2_S in the human body remain unclear and most of the mechanisms of action underlying the effects of H_2_S have yet to be fully elucidated. The primary purpose of this review was to provide an overview of the role of H_2_S in the human body and to describe its beneficial effects.

## 1. Introduction

Hydrogen sulfide (H_2_S) is a poisonous gas that is a toxicant in most organs in the human body. It acts as a gaseous signaling molecule and chemical reagent involved in many physiological processes, including the pathogeneses of various diseases such as neurodegenerative disease, heart failure, and diabetes [1, 2]. As a result, the beneficial roles of this compound were neglected for many years due to its toxic nature. However, in recent years, the benefits of gasotransmitters such as nitric oxide (NO) and carbon monoxide (CO) have acted as a fillip to investigating the benefits of H_2_S [3]. Furthermore, a reevaluation of the endogenous levels of H_2_S confirmed its existence and advantages in mammalian tissues [4].

H_2_S is a sulfur analog of water and, due to its weak intermolecular force, exists in a gaseous form that is colorless but has an offensive odor [5]. At a pH of 7.4 in the mammalian body, one-fifth of the total H_2_S subsists in an undissociated form, with the remaining content existing as hydrosulfide anions (HS^−^) and sulfide (S^2−^). The high lipid solubility of H_2_S allows it to easily penetrate the plasma membrane of cells in its undissociated form [6] but it remains unclear whether this undissociated form is physiologically pertinent [7]. Various studies performed on rat, human, and bovine brain tissues have determined that H_2_S is present at levels of up to 50–160 *μ*mol/L in tissues and that sodium hydrogen sulfide (NaHS) is one of the physiological donors of H_2_S [8].

Although it is known that H_2_S protects nerves from oxidative stress, saves photoreceptor cells in the retina from light-induced degeneration, regulates endoplasmic reticulum stress, and defends the kidneys from ischemic reperfusion injury [9], the role of H_2_S in the central nervous system (CNS) has attracted a lot of attention over the past few decades. The neuromodulation and neuroprotection of nerve cells are a common feature of H_2_S [9] and its enzymes exert effects in a variety of diseases such as Parkinson's disease (PD), Alzheimer's disease (AD), Down's syndrome, cerebral ischemia, and Huntington's disease [10, 11]. Additionally, following injury in the peripheral nervous system (PNS), peripheral nerves undergo Wallerian degeneration and macrophages are recruited into the distal nerve pump [12]. The regeneration of these injured peripheral nerves is associated with the demyelination, dedifferentiation, and proliferation of Schwann cells, which takes place serially. Recently, the possible involvement of H_2_S in demyelinating disorders and nerve degenerative disorders has also been suggested [13, 14].

Thus, the present review focuses on the physiological roles of H_2_S in different systems and organs, including the CNS, and in peripheral nerve degeneration and regeneration. A consensus regarding findings in these areas will aid in the determination of appropriate avenues for H_2_S in research investigating neural regeneration and the treatment of neurodegenerative diseases and may lead to potential therapeutic strategies that employ H_2_S.

## 2. Biosynthesis of H_2_S

Two pyridoxal-5′-phosphate- (PLP-) dependent enzymes present in mammalian tissues, cystathionine *β*-synthase (CBS; EC 4.2.1.22) and cystathionine *ϒ*-lyase (CSE; EC 4.4.1.1), are primarily responsible for the biosynthesis of H_2_S from L-cysteine (L-Cys) [15, 16]. H_2_S can be produced by other pathways as well. Cysteine first reacts with ketoacids to form 3-mercaptopyruvate via the catalytic action of cysteine aminotransferase (CAT; EC 2.6.1.3) and then 3-mercaptopyruvate is desulfurated by 3-mercaptopyruvate sulfurtransferase (MST; EC 2.8.1.2) to form H_2_S [17]. H_2_S is mostly synthesized by cystathionase in the liver, kidneys, enterocytes, and vascular smooth muscle cells, by CBS in the brain, and by MST in cardiac tissues [18]. Recently, it was reported that dihydrolipoic acid (DHLA) and thioredoxin are the endogenous reducing substances that cause MST to release H_2_S [16] and that CBS is the catalytic agent responsible for the condensations of cysteine and homocysteine, which produces H_2_S and cystathionine via a *β*-replacement reaction [19, 20]. The distributions of CBS and CSE in mammalian tissue are different. A cardiovascular study determined that CSE plays a major role in producing H_2_S under normal physiological conditions [21] while another study found that the MST pathway mainly contributes to its production in the brain and that this pathway is regulated by intracellular calcium (Ca^2+^) in a concentration-dependent manner [16, 22, 23].

**Figure 1:**
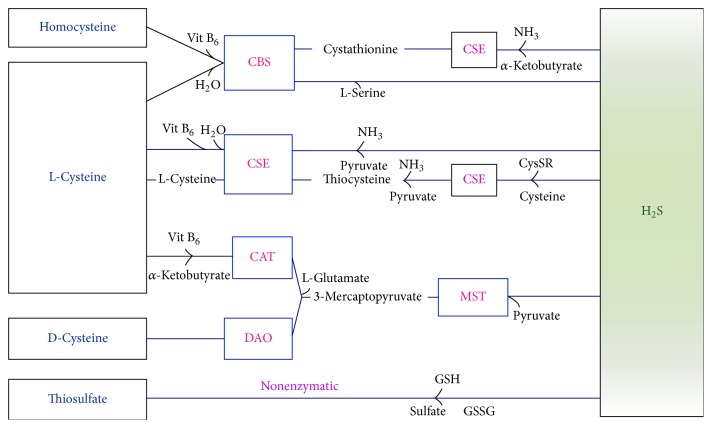
Biosynthetic pathways and the transformation of H_2_S in the mammalian body. H_2_S is synthesized in the mammalian body via both enzymatic and nonenzymatic pathways but the nonenzymatic pathway accounts for only a small portion of its production. CBS and CSE both use PLP and vitamin B_6_ as cofactors. In the presence of cysteine and more so in the presence of homocysteine, CBS catalyzes the production of H_2_S, and the condensations of homocysteine and serine are the most recognized reactions catalyzed by CBS. CSE uses L-cysteine as the substrate to form two gases, H_2_S and NH_3_, as well as pyruvate. MST and CAT produce H_2_S and pyruvate from 3-mercaptopyruvate, which is formed from L- or D-cysteine, and *α*-ketoglutarate, which is produced by CAT. Thiosulfate nonenzymatically produces H_2_S and all essential components of this nonenzymatic path are present in vivo. Thiosulfate can be converted into sulfite in the liver, kidney, or brain tissues via thiosulfate reductase or by thiosulfate sulfurtransferase in the liver. H_2_S is also released from thiosulfate and persulfides [8, 30].

A recent study used Western blot and immunohistochemistry analyses to investigate DAO expression and methylene blue assays to assess H_2_S biosynthesis and revealed that H_2_S is also produced from D-cysteine via the enzyme D-amino acid oxidase (DAO) [24]. The H_2_S production pathway that uses D-cysteine primarily operates in the cerebellum and kidney and, thus, was termed the DAO/MST pathway [25, 26]. Although DAO is confined to peroxisomes and MST is located in the mitochondria, these factors exchange numerous enzymes and metabolites [16]; these findings provide strong support for calling this pathway the DAO/MST pathway. Interestingly, the production of H_2_S via DAO is subdued by indole-2-carboxylic acid but this effect was not seen with MST [26]. A nonenzymatic pathway for the production of H_2_S also exists and involves the reduction of elemental sulfur to H_2_S by reducing equivalents procured via the oxidation of glucose [27]. Either hyperglycemia or the escalation of oxidative stress conditions can nurture the production of H_2_S via this nonenzymatic route in mammalian erythrocytes [8]. The endogenous production and metabolism of H_2_S and its aforementioned biosynthetic and transformation pathways are illustrated in Figure 1.

It is believed that two possible mechanisms can explain the release of H_2_S. First, after its production, H_2_S may be liberated by the enzymes involved in its synthesis and, second, H_2_S may be stored and released in response to physiological needs in mammalian tissue [28]. H_2_S is released from acid-liable sulfur (sulfur atom in iron-sulfur complex) under acidic conditions, while under alkaline conditions bound sulfane-sulfur comes into play; the favorable pH range for its release under these conditions is approximately 5.4 and 8.4, respectively [29]. The discovery of the DAO/MST pathway suggests that additional H_2_S biosynthetic pathways may be revealed in the future.

## 3. Interrelationships with Other Gasotransmitters

Although H_2_S, NO, and CO have similar molecular targets and cellular actions, these compounds also have a tendency to compete with each other. For example, the gasotransmitters CO and NO have particular relationships with CBS [8]. After the initial demonstration of the physiological roles of H_2_S in 1996 [31], it took 5 more years to determine that NO can bind to CBS and can impede enzymatic activity and that CBS has a high affinity for CO [32]. However, the exact mechanisms underlying these relationships remain unknown. NO also modulates the endogenous production of H_2_S in smooth muscle cells and vessels [33]. Previously, it was thought that the signaling pathways and functional mechanisms associated with H_2_S and NO were autonomous, but their combined effects on angiogenesis and vasorelaxation have complicated this issue for researchers. If endothelial NO synthase (eNOS) is blocked, then it fully rescinds the angiogenic effects of H_2_S whereas the silencing of H_2_S significantly attenuates the angiogenic effects of NO [34]. Additionally, both H_2_S and NO activate protein kinase G, as well as its cellular signaling, and 1-H-[1,2,4]oxadiazolo[4-3,-a]quinoxalin-1-one (ODQ), which is a soluble guanylyl cyclase inhibitor that decreases both H_2_S- and NO-induced angiogenesis [35].

Under severe conditions such as heart inflammation and heart failure events, H_2_S and NO cooperate and communicate with each other to stimulate thiol-sensitive compounds that produce unambiguous positive outcomes for inotropic and lusitropic heart abnormalities [36]. Ex vivo aortic explants of wild-type and CSE-knockout (CSE-KO) mice were used to explore the possible interaction between H_2_S and NO and revealed that CSE-KO mice exhibit a significant decrease in vascular neogenesis [37]. The authors also proposed that the interaction of these gasotransmitters stimulates endothelial cell proliferation. The administration of H_2_S to rats with clinical symptoms of hypoxic pulmonary hypertension has also been shown to increase plasma CO concentrations [38]. CO and H_2_S act on the same target but have opposite patterns of outcome. For example, coevoked channel activation is completely suppressed by potassium cyanide (KCN; 1 mM) but there are no consequences during H_2_S-induced channel activation [39]. Thus, the precise nature of the relationship between these gasotransmitters in various physiological pathways remains unclear and requires further investigation. However, even if H_2_S, NO, and CO compete with each other, their interactions are likely to result in beneficial effects on mammalian physiology.

## 4. Effect of H_2_S on the CNS

H_2_S has a proven neuromodulatory role in the protection of neurons from oxidative stress, as evidenced by its inhibition of hypochlorous acid-mediated oxidative damage [40] and ONOO^−^-mediated protein nitration and cytotoxicity [41] in neuroblastoma cells. Recent studies have also established that H_2_S plays an important role in the upregulation of the GABA *β*-receptor at both pre- and postsynaptic sites [42]. Astrocytes and microglial cells play important roles in the regulation of brain pH levels, neurotransmitter levels, and neuronal excitability [43] while microglia are also associated with the progression of neuronal diseases such as AD [44] and PD [45]. H_2_S evokes Ca^2+^ waves in astrocytes that trigger a Ca^2+^ influx via its channels in the plasma membrane [7] and also reversibly increases Ca^2+^ levels in microglia in a dose-dependent manner [46].

**Figure 2:**
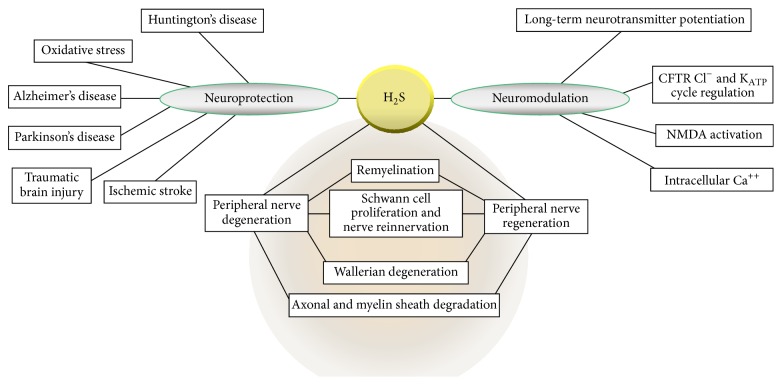
Therapeutic targets and possible physiological functions of H_2_S. Enough experimental evidence has been collected to prove the prominent role of H_2_S in normal pathophysiology. Therefore, many therapeutic targets exist for H_2_S in mammalian body; its roles in neuroprotection, neuromodulation, and antiproliferation, as well as its functions during peripheral nerve degeneration and regeneration, are widely appreciated.

In addition to regulating Ca^2+^ homeostasis, H_2_S may also be involved in long-term potentiation (LTP) and the modulation of various neurotransmitters [5]. H_2_S facilitates hippocampal LTP via the activation of N-methyl-D-aspartate (NMDA) receptors as well as the phosphorylation of these receptors by protein kinase A (PKA) [47] and regulates intracellular Ca^2+^ level in astrocytes and hippocampal slices [10]. H_2_S also safeguards neurons by controlling endoplasmic stress via the balancing of membrane potentials and the activation of K_ATP_ and cystic fibrosis transmembrane conductance regulator chloride (CFTR Cl^−^) channels [9]. Most neurons in the nucleus solitarius, which are believed to play a role in the cardiovascular system, are depolarized by H_2_S [48]. Additionally, a donor of H_2_S, NaHS, is linked to the inhibition of apoptosis, decreases of edema in the brain, and the amelioration of cognitive dysfunction, which could attenuate early brain injury development due to subarachnoid hemorrhage via several mechanisms [49]. The major roles and possible therapeutic targets of H_2_S are illustrated in Figure 2.

Thus, it can be concluded that H_2_S exerts protective and modulatory effects on nerve cells, either cooperatively or independently. Although the physical and chemical properties of H_2_S remain elusive, its roles in these processes have become more clearly defined and most of its mechanisms of action are understood.

### 4.1. H_2_S and PD

PD is primarily characterized by cognitive deficiencies resulting from changes in the nucleus basalis of Meynert and the cerebral cortex and the continuous loss of dopaminergic neurons in the mesencephalon [50]. H_2_S inhibits oxygen consumption and 6-OHDA-evoked nicotinamide adenine dinucleotide phosphate (NADPH) oxidation and activates microglial cells in the midbrain which, in turn, lead to the accumulation of proinflammatory factors in the subcortical part of the forebrain [51]. This is the primary mechanism by which H_2_S decreases the chances of further neuronal injury and degeneration [52]. The neuroprotective role of H_2_S has also been demonstrated in experimental rat models of neurotoxin-induced PD [50]. The advantageous effects of H_2_S are due to its activation or suppression of different protein kinases, such as PKC, PI3K/Akt, p38, JNK, and the ERK-MAPKs [53], which decrease oxidative stress and inflammation and exert antiapoptotic actions.

Although L-Dopa is the most commonly used drug for the treatment of PD due to its ability to maintain dopamine levels, it cannot block or reverse the progression of PD. Additionally, long-term L-Dopa therapy may lead to neurodegeneration [54] and dyskinesia [55] in and of itself. H_2_S stimulates glutamate transporter functioning and leads to direct sulfa hydration via the ERK/MAPK pathway, which attenuates the production of reactive oxygen species and decreases oxidative stress [56, 57]. Thus, it can be concluded that L-Dopa and H_2_S may be more effective for the treatment of PD when used in combination. A mouse model of PD constructed using 1-methyl-4-phenyl-1,2,3,6,-tetrahydropyridine (MPTP) plus probenecid injections results in the destruction of dopaminergic neurons but the administration of H_2_S increases the survival rate of neurons and is protective against MPTP-induced toxicity [58]. Thus, H_2_S not only protects peripheral tissues but also effectively treats neurological damage related to PD. Recently, it was shown that drinking coffee and inhaling cigarettes can inhibit monoamine oxidase (MAO) [59], which suggests that there is a lower risk of PD in coffee drinkers and smokers. Cakmak found that coffee contains* Prevotella*-derived H_2_S and that H_2_S is a well-documented constituent of cigarette smoke.

### 4.2. H_2_S and AD

AD, which is one of the most familiar types of dementia, is caused by activated microglia and increases in neuritic plaques carrying the *β*-amyloid protein [60]. This neurodegenerative disease has been exhaustively researched because it affects the cortex and hippocampus and leads to severe cognitive dysfunction [61]. The etiology of AD is multifactorial and presumably includes a number of distinctive etiopathogenic mechanisms [62]. CBS is thought to be the main source of H_2_S in the brain. In 1996, it was first shown that S-adenosylmethionine, which is a CBS activator, is significantly reduced in subjects with AD [63]. Moreover, the severity of AD is related to altered levels of H_2_S [64] because the pathological state of AD in the human body has been associated with lower levels of endogenous H_2_S and the accumulation of homocysteine in the brain [65, 66]. Several series of in vitro and in vivo experiments demonstrated the role of H_2_S in the promotion of cell growth and preservation of mitochondrial function [67] as well as in the retardation of oxidative stress factors such as amyloid beta peptides (A*β*), malondialdehyde (MDA), hypochlorite (HOCI), and 4-hydroxy-2-nonenal (4-HNE) [68, 69]. In adult male Wistar rats without serious signs of H_2_S toxicity, spa water with excessive amounts of H_2_S has the ability to improve cognitive processes by decreasing A*β* deposits and targeting the APP, PST, and ON/4R-tau isoforms [70]. Additionally, NaHS, which is a donor of H_2_S, retards protein oxidation and lipid peroxidation in the neuroblastoma cells of AD patients [71].

Cerebral atrophy, seizures, and intellectual disabilities can be caused by the autooxidation of homocysteine [72, 73] and hyperhomocysteinemia has been identified in brains of AD patients [74]. H_2_S protects against and reduces homocysteine-induced toxicity and oxidative stress through its antioxidant properties in the adrenal medulla (PC12 cells) and vascular smooth muscle cells of rats [75, 76]. Synaptic dysfunction and vascular inflammation are also believed to play crucial roles in the pathogenesis of AD [77]. Recent analyses of the expressions of mRNA and synaptic proteins in C57BL/6J wild-type male mice clearly demonstrated that plasma homocysteine-induced alterations in learning and memory processes were associated with synaptic remodeling in the hippocampus [78]. Thus, H_2_S can influence synaptic remodeling. Vascular dementia (VD) is another common neurodegenerative disorder that, much like AD, is caused by cerebral ischemia. H_2_S modulates oscillatory coupling in the hippocampus and may represent a possible molecular mechanism underlying the changes in VD patients [79].

Although neurodegenerative pathologies like AD and PD do not initially involve inflammation, various experimental findings suggest that the inflammatory responses of macrophages, microglia, and astrocytes contribute to the progressions of both diseases [14]. The relevance of the CBS, CSE, MST, and CAT enzymes in the development of AD and PD is still unexplained and direct evidence supporting the potential advantages of H_2_S as a therapeutic strategy for these diseases is unavailable.

### 4.3. Other CNS Diseases

Various experimental studies have correlated the effects of H_2_S in different pathological states of the human body. Ischemic stroke increases tissue levels of H_2_S in the cerebral cortex [80] while H_2_S has been shown to protect the embryonic brain against ischemia-reperfusion injury [81]. A rat model of febrile seizure is associated with elevated plasma levels of H_2_S, and Down's syndrome is known to cause the overaccumulation of H_2_S in the brain [42]. Similarly, there are increased total plasma homocysteine levels in patients with Huntington's disease and CBS deficiencies lead to homocystinuria [50]. Additionally, H_2_S reverses learning and memory problems caused by damage to the hippocampus [82, 83].

## 5. H_2_S and the PNS

Even if the most important roles that glial cells play involve the physical and metabolic support of neurons via the maintenance of the extracellular environment, these support cells are often referred to as “glial culprits” because the CNS lacks the ability to regenerate itself, even after injury [84]. On the other hand, Schwann cells in the PNS are best known for their roles in supporting nerve regeneration, conducting nerve impulses along axons, and modulating neuromuscular synaptic activity and nerve development [85]. The possible roles of H_2_S in peripheral nerve degeneration and regeneration are discussed below and supported with experimental evidence.

### 5.1. H_2_S in Peripheral Nerve Degeneration

Based on the degrees of damage in the nerve and surrounding connective tissue, peripheral nerve damage may be classified as neurapraxia, axonotmesis, and neurotmesis, with the latter being a severe type of peripheral nerve injury [86]. As stated above, nerve regeneration after injury is possible in the PNS and involves major events such as Wallerian degeneration, axonal degeneration, remyelination, axonal regeneration, and nerve reinnervation. H_2_S plays vital roles throughout this process. Axonal regeneration and remyelination begin in the distal pump of injured peripheral nerves and involve axonal degeneration and the degradation of the myelin sheath of Schwann cells, which is termed Wallerian degeneration [87].

The effects of H_2_S on peripheral nerve degeneration and regeneration may be best explained by recent ex vivo experiments using the sciatic nerves of mice. In these experiments, Park et al. [12] utilized N-ethylmaleimide (NEM), which is an inhibitor of all cysteine peptidases, to inhibit the production of H_2_S during Wallerian degeneration and found that NEM inhibits not only CSE but also the basal expression of MST. Based on analyses of several markers of Schwann cell dedifferentiation and proliferation, these authors concluded that H_2_S signaling has great value after peripheral nerve injury regarding myelin fragmentation, axonal degradation, and Schwann cell dedifferentiation and proliferation. Additionally, these findings demonstrated that H_2_S production influences transcriptional regulation in Schwann cells during Wallerian degeneration. A similar study inhibited these processes via NEM or through several Schwan cell dedifferentiation or immaturity markers including lysosomal associated membrane protein 1 (LAMP1), neurotrophin receptor p75 (p75^NTR^), the protein coded by JUN gene (c-jun), and phospho-ERK 1/2 (p-ERK1/2) [15]. Jung and Jeong found that these types of inhibition decreased myelin ovoid fragmentation, axonal degeneration, and Schwann cell dedifferentiation, demyelination, and proliferation through neurotrophin receptors, the MAPK pathway, lysosomal protein degradation, and transcriptional regulation.

The use of these markers in current research has garnered much attention. For example, the LAMP1 marker is related to the degeneration of peripheral nerves, including the nonuniform morphology of myelinated axons, in aged individuals [88]. The function of p75^NTR^ is associated not only with cell death and survival but also with the maintenance of axonal elongation and the release of neurotransmitters between sympathetic neurons and cardiac myocytes [89]. Furthermore, the reexpression of p75^NTR^ has been identified in various pathological scenarios, including neurodegenerative disorders [90]. Due to its roles as a protein and a component of a transcription factor, the inactivation of c-jun in mice resulted in delays and/or the failure to properly complete the regeneration procedure and an enhancement of neuronal death [91]. Although the molecular pathways are not yet fully understood, the absence of c-jun causes the complete downregulation of neurotrophic genes such as artemin (Artn), brain-derived neurotrophic factor 1 (BDNF-1), glial cell-derived neurotrophic factor (GDNF), leukemia inhibitory factor (LIF), and nerve growth factor (NGF). These findings suggest that neurotrophins may be a potential target for the treatment of various neuropathies. Studies of the molecular mechanisms involved in those processes explained above have revealed the capability of Schwann cells to regenerate axons and to rescue motor neurons following nerve injury [92].

However, many issues relevant to this process remain unexplained, including the relationships between neurotrophic factors and H_2_S, H_2_S production, and remyelination that is associated with the extracellular matrix (ECM) protein, as well as intracellular regulators, hormones, and transcriptional regulators that involve H_2_S [15]. Other than H_2_S, the ubiquitin proteasome system (UPS) is another factor that is essential for the regenerative functions of peripheral nerves after injury [93, 94].

### 5.2. H_2_S in Peripheral Nerve Regeneration

Other issues associated with the role of H_2_S in peripheral nerve regeneration involve the responses of Schwann cells in H_2_S production. Schwann cells are believed to be an important factor in the regeneration process because peripheral axons have no function as H_2_S producers during Wallerian degeneration [12]. Nerve injury leads to the dedifferentiation of Schwann cells from myelinated cells to immature undeveloped cells [15] and, as discussed in Section 5.1, decreases in H_2_S production attenuate changes in markers of dedifferentiation. Thus, it is important that reductions in H_2_S production nurture both the regeneration of axons and the remyelination during the last stage of Wallerian degeneration. Immature undeveloped Schwann cells undergo multiplication and reach the target, injured organ, which is when the possible beneficial effects of H_2_S come into play. Remyelination is another important process involved in nerve regeneration whereby Schwann cells are able to flourish due to myelination and the inhibition of H_2_S production (via transcriptional regulation through krox20 and c-jun), which is fundamental to this stage of regeneration [15].

Despite the above findings, very few studies have attempted to analyze the roles that H_2_S plays in peripheral nerve degeneration and regeneration. Although our research group is currently conducting studies to identify the core effects of H_2_S, the existing literature strongly supports the importance of H_2_S in nerve degeneration and regeneration.

## 6. Other Functions of H_2_S

The exciting evolution of H_2_S in the field of neuroscience has revealed its importance not only in the PNS but also in various physiological and pathological conditions. For example, endogenous levels of H_2_S have been shown to influence exogenous H_2_S during cell apoptosis [95]. Additionally, the antiproliferative and proapoptotic effects of H_2_S have a significant influence on various disorders such as vascular graft occlusion, atherosclerosis, and neointimal hyperplasia [96]. H_2_S also regulates glutathione levels by enhancing the hustle of the cysteine/glutamate antiporter at the cellular level, which directly neutralizes free radicals and reactive oxygen compounds and balances the levels of vitamins C and E in their reduced form [97]. The acute inhalation of H_2_S protects lungs from injuries induced by ventilators and relaxes pulmonary vascular tissue but epidemiological data suggest that the long-term exposure to even low levels of H_2_S can cause bronchial hyperresponsiveness [8].

Relative to other parts of the body, the gastrointestinal tract (GIT) contains the largest amount of H_2_S, where it subsidizes the homeostatic control of GIT mucosal defenses and repairs damage [31]. However, the signaling pathways of H_2_S in the GIT remain unclear. Other beneficial effects of H_2_S on translation and transcription include the control of endoplasmic reticulum stress and activating the unfolding protein response. During these processes, CSE increases H_2_S production and ultimately restores endoplasmic reticulum homeostasis via the sulfa hydration reaction [98]. Additionally, type 1 diabetes is believed to involve the overproduction of H_2_S [18], and H_2_S may be helpful for patients with erectile dysfunction due to its involvement in the relaxation of the smooth muscle that causes the erection of the penis [99]. Recently, six fatal cases of H_2_S poisoning were reported during an attempt to unblock a wastewater cistern in which the primary reason for death was H_2_S aspiration and because it is a mediator and regulator of various physiological conditions, H_2_S can have serious toxic effects [100].

The roles of H_2_S in insulin resistance syndrome and regional ischemic damage, as well as its dose-dependent relationship with methylglyoxal (MG) in vascular smooth muscle cells, also require explanation [3]. Because it is an antioxidant bulwark, H_2_S has the capacity to sense chromaffin cells and chemoreceptors [101] but it has yet to be determined how H_2_S increases local blood perfusion and/or ventilation [102]. Further justification is needed for the production of H_2_S via MST under physiological conditions because this process requires full alkaline conditions [103].

## 7. Therapeutic Prospect and Potential of H_2_S

Almost two decades of research on H_2_S unfastened series of positive outcomes and its potential is expanding every day. Molecular mechanisms of H_2_S are being uncovered and various types of molecular and sulfa hydration targets are on the phase of identification which may lead us to reveal its biological activities [10]. Several in vivo and in vitro studies on AD and PD model have already proved their therapeutic effectiveness for treatment [40, 68, 104, 105]. Surprisingly, inhaled H_2_S has found to have protective effect against neuropathic pain and brain edema which has also made researchers think seriously in this topic because we can develop our opinion and research arena on hydrogen sulfide donor compounds against neuropathic pain and brain edema [106, 107]. Even though H_2_S has to deal with its own double face (toxic and protective) attitude which has pushed it into several controversies, it is recently found that various ion channels in multiple systems and organs advocate the protective role of H_2_S [108]. Because of the overall effect, H_2_S-releasing drugs are now under clinical trial after their verified effectiveness in animal model. This trend of clinical trial on finding H_2_S donors or H_2_S-releasing drugs can be a very big breakthrough for the treatment of several diseases which are almost incurable till date [109]. H_2_S-releasing derivatives of mesalamine [110] and diclofenac [111] have already been shown to decrease inflammatory disease and gastric hemorrhagic lesions compared to original drugs and some possibility for the treatment of injured brain after subarachnoid hemorrhage (SAH) via H_2_S is also a great progress [112] because another study demonstrated that it reduces the level of reactive oxygen species and lipid peroxides malondialdehyde following SAH [113]. Recent studies on the effect of H_2_S on brain synaptic remodeling and its role in GABA-mediated and glutamate neurotransmission have even increased its prospective potential in physiological standpoint [114]. Even with the difference between community and industry based results, the effect of environmental exposure to H_2_S on CNS has exposed its novel and diverse role [115]. Neuroprotective role of H_2_S after traumatic brain injury has also proven its potentiality and efficacy for other CNS related diseases [116]. Thus, it can be summarized that future prospect of H_2_S in development of new therapeutic strategy is wide and bright.

## 8. Concluding Remarks

An adequate amount of evidence has been gathered in support of H_2_S as a gasotransmitter and modulator in mammalian tissue, particularly in the nervous system. H_2_S is principally produced in the liver, kidney, enterocytes, and vascular smooth muscle cells via cystathionase and partially produced in cardiac tissue by MST. It is catabolized in mitochondria by thiosulfate reductase, and thiosulfate in the urine may be used as a marker of H_2_S biosynthesis. H_2_S is capable of both suppressing and promoting inflammation but it remains unclear how these proinflammatory and anti-inflammatory activities can be enhanced or attenuated. The role of H_2_S in the Schwann cell response to peripheral nerve injury has been well established by experimental evidence, and the importance of H_2_S signaling during Wallerian degeneration, where it broadly affects Schwann cell dedifferentiation and proliferation, has been repeatedly demonstrated.

The bidirectional relationship between H_2_S and NO is another area of particular research interest and therapeutic potential. Experimental studies using inhibitors or donors of endogenous H_2_S have produced clear evidence of its effects but clinical studies that can define its role in the treatment of diseases are necessary. Pharmaceutical companies should accelerate their attempts to formulate, design, and produce H_2_S-releasing drugs with sustained/controlled release functions as they are likely to be highly innovative for the treatment of neurodegenerative diseases. Thus, therapeutic prospects of H_2_S signaling for patients with neurodegenerative disease and demyelination disorders are imminent. However, the uncontrollable release, unidentified byproducts, and unclear mechanisms of release and action represent major issues related to the research and clinical use of H_2_S. Although it is currently a time of great interest and excitement regarding the potential of H_2_S in medicine and biology, many hypotheses regarding H_2_S have yet to be supported and many issues remain unanswered. Even though various novel molecular targets have been identified and research is hastily expanding, biomedical research into this gasotransmitter is still in its initial stages.
